# Historical review of clinical vaccine studies at Oswaldo Cruz Institute and Oswaldo Cruz Foundation - technological development issues

**DOI:** 10.1590/0074-02760140346

**Published:** 2015-02

**Authors:** Reinaldo de Menezes Martins, Cristina de Albuquerque Possas, Akira Homma

**Affiliations:** Conselho Político e Estratégico, Bio-Manguinhos-Fiocruz, Rio de Janeiro, RJ, Brasil

**Keywords:** clinical studies, vaccines, technological development, innovation, regulatory barriers

## Abstract

This paper presents, from the perspective of technological development and production, the results of an investigation examining 61 clinical studies with vaccines conducted in Brazil between 1938-2013, with the participation of the Oswaldo Cruz Institute (IOC) and the Oswaldo Cruz Foundation (Fiocruz). These studies have been identified and reviewed according to criteria, such as the kind of vaccine (viral, bacterial, parasitic), their rationale, design and methodological strategies. The results indicate that IOC and Fiocruz have accumulated along this time significant knowledge and experience for the performance of studies in all clinical phases and are prepared for the development of new vaccines products and processes. We recommend national policy strategies to overcome existing regulatory and financing constraints.

Clinical studies are crucial for the development and registration of new products and constitute today a structured process, mandated by strict legislation involving a growing number of participants, in a stepwise strategy.

The Oswaldo Cruz Institute (IOC) and the other technical units which constitute the Oswaldo Cruz Foundation (Fiocruz) are recognised as very important institutions for basic science and biological and technological research on tropical diseases in Brazil. These institutions have a long tradition of clinical studies which have proven to be vitally connected to the prevention of infectious diseases of public health importance for Brazil and other countries. In this paper, we review and analyse these studies, occurring over a period exceeding seven decades, from the perspective of technological development (TD). The understanding, in a historical sense, of the evolutionary stages of these clinical studies will hopefully provide a better understanding of the processes that were involved and may help policy and decision-makers to conceive of new alternatives and create possibilities for the design of new studies in the future.

## MATERIALS AND METHODS

For the selection of the clinical studies we adopted the following criteria for inclusion: (i) studies conducted in human beings; (ii) prospective; (iii) vaccination as the basic intervention; (iv) longitudinal and individual follow-up of participants; (v) published in scientific medical journals; (vi) conceived and conducted according to ethical and legal criteria for clinical research in human beings, with tolerance to the absence of formal ethical and regulatory evaluations regarding the older studies; (vii) conducted with participation of at least one unit or professional of Fiocruz/IOC.

These restrictive criteria, besides being conceptually acceptable, met the requirement to limit the scope of the research within an acceptable range. Studies which did not meet these criteria were excluded. These included retrospective studies, clinical-epidemiological studies, seroepidemiological studies, pharmacovigilance studies and observational studies. Although the latter did not fit into the classical model of clinical studies, some of them could be classified as clinical studies, in a broader sense definition.

For studies conducted at the origin of IOC, which are outstanding and part of its history, these criteria were not strictly applied, which is justifiable, considering that the legislation on clinical studies came later. However, if they are not formally perfect, they have been conducted ethically, with the best science and methodology available at the time.

The search for papers was done by databases, including PubMed, from the National Center for Biotechnology Information, National Institutes of Health, United States of America, and LILACS, the Latin-American database from BIREME-Regional Library of Medicine, from Pan American Health Organization (PAHO)/World Health Organization (WHO). However, most papers were found through personal archives, consultations with colleagues, some reference books on the history of vaccines (Benchimol 2001, Artenstein 2010, Plotkin 2011) and other means, in a process with considerable degree of serendipity in chance encounters.

To recover original papers, we used, besides PubMed and LILACS, the SciELO database, Capes Periodicals Gateway and the libraries of the National School of Public Health, Bio-Manguinhos/Fiocruz and Mourisco Castle. Photocopies were also obtained from the Hinari Programme, from WHO and Oxford Journals.

We use terms "clinical trial" and "clinical study" interchangeably.

The period of study extended up to the year 2013 (Martins 2014).

## RESULTS

Tables I-IV show, for each study, its rationale and its basic findings. Table V shows the number of clinical studies by kind of vaccine (viral, bacterial or parasitic) and its utilisation [commercial or by the National Immunisations Program (NIP)].

**Table:** TABLE I Synopsis of studies with yellow fever (YF) vaccine in chronological order of publication

Reference	Rationale of the investigation	Comments
Soper and Smith (1938)	Use of substrain 17E in human beings with human immune serum and hyperimmune serum from goats and monkeys.	Reports of icterus and serum reactions.
Smith et al. (1938)	Large scale vaccination with substrain 17D without animal serum.	Establishment of YF vaccine production at Oswaldo Cruz Institute.
Fox et al. (1942a)	Investigate icterus outbreak after YF vaccination.	Recommendation for elimination of human serum from YF vaccine production and establishment of seed lot system.
Fox et al. (1942b)	Investigate outbreak of encephalitis after vaccination with substrain 17D-NY 104.	Suspension of use of substrain 17D-NY 104.
Fox and Cabral (1943)	Duration of immunity after YF vaccine, with several substrains and in several age groups.	Immunity persists after YF vaccine during at least four years in adults, lower immune responses in children less than 10 years of age.
Fox et al. (1943)	Choice of a new seed lot, dose-response, alternatives for vaccine administration (subcutaneous, intramuscular, intradermal).	This study was done before a previous study (Fox et al. 1942a), but published later. Human serum still used on the vaccine. A new seed lot was chosen, the substrain 17D-NY 104, that proved later to be neurotropic.
Fox et al. (1948)	Additional investigations on duration of immunity after YF vaccine.	Additional indications of lower seroprotection and duration of immunity in children.
Groot and Ribeiro (1962)	Long term indications of seroprotection for 17 years.	This study was done in a region previously vaccinated with the 17D-NY 104 substrain.
Lopes et al. (1988)	Dose-response study.	A dose of 1000 plaque forming units (or its equivalent in LD_50_) is more than enough for protection against YF.
Stefano et al. (1999)	Investigate the interference between measles vaccine and YF vaccine.	There is no interference between measles vaccine and YF vaccine.
Freire et al. (2002)	Investigate immune response and duration of immunity after YF vaccine 17DD at egg-passage 43.	High levels of antibodies before YF vaccine inhibit the immune response. Persistence of immunity at 10 years after YF vaccination is questioned.
Camacho et al. (2004)	Immunogenicity of vaccine from new seed lot (17DD-013Z) compared to previous seed lot (17DD-102/84), World Health Organization (WHO) vaccine (17D-213/77) and placebo.	Advances on methodology and adherence to National Health Council norms. The new seed lot has adequate immunogenicity.
dos Santos et al. (2005)	Interspecific and specific immune response in vaccinated and revaccinated subjects.	YF vaccine induces immune response with activation and memory of humoral and cellular arms.
Camacho et al. (2005)	Reactogenicity of vaccine produced with new seed lot compared to previous seed lot (17DD-102/84), WHO vaccine (17D-213/77) and placebo.	The new seed lot is safe.
dos Santos et al. (2007)	Study of lymphocyte subpopulations according to the time of processing of blood samples.	Flow cytometry by the lysis method may be done at times 0, 24 or 48 h after blood collection.
Martins et al. (2007)	Study of lymphocyte subpopulations in 10 healthy adults primovaccinated with 17DD vaccine.	The immune response is complex, activation and modulation seem to occur at the same time.
Santos et al. (2008)	T helper (TH)1/TH2 cells immune responses in 12 healthy adults vaccinated against YF.	After peripheral blood mononuclear cells ex vivo stimulation with 17DD vaccine, there is increase in interferon (IFN)-γ and interleukin-4, reaching maximum levels 15 days after vaccination.
Martins et al. (2008a)	Phenotypical response of innate immunity in 10 adults after YF vaccine.	There is a balanced response of activation and modulation, studied in neutrophils, eosinophils, monocytes, natural killer (NK) and NKT cells.
Neves et al. (2009)	Expression of toll-like receptor (TLR) and activation of NK cells in eight healthy adults vaccinated against YF.	NK cells are activated soon after vaccination against YF. IFN-γ is increased on day 15 after vaccination.
Melo et al. (2011)	Duration of immunity after YF vaccine.	17DD vaccine protects for at least 10 years, but with decreasing titres. Small sample.
Silva et al. (2011)	Investigate the interference between YF vaccine and measles-mumps-rubella (MMR) vaccine when applied simultaneously.	Negative and reciprocal interference of YF, rubella and mumps antigens.
Luiza-Silva et al. (2011a)	Cytokines in children from nine-43 months of age vaccinated against YF.	Cytokine signatures show proinflammatory cytokines on subjects who sero-convert (SC) to YF vaccine and regulatory on non SCs. Revaccinated one year later, there was SC of previously non SCs with a proinflammatory pattern.
Luiza-Silva et al. (2011b)	Cellular sources of cytokines in 10 healthy adult vaccinated against YF.	Pattern of activation and modulation. Production of IFN-γ on day 7 by NK cells and on days 15 and 30 by T CD4+ (TH) cells.
Campi-Azevedo et al. (2012)	Cytokine profile in 80 children from nine-12 months of age, vaccinated with YF 17DD or 17D-213/77.	The YF reference vaccine from WHO has a immune response similar to the 17DD vaccine from Bio-Manguinhos/Oswaldo Cruz Foundation.
Martins et al. (2013b)	Dose-response to YF vaccine in healthy young male adults.	YF vaccine from Bio-Manguinhos is so immunogenic in doses = 587 IU as in the usual dose of about 27,476 IU.
Melo et al.(2013)	Memory after YF vaccine. Blood collected before vaccination two months and four years after vaccination.	YF promiscuous antigens may be better immunogens.

Source: Martins (2014).

**Table:** TABLE II Synopsis of studies with other viral vaccines in chronological order of publication

Vaccine	Reference	Rationale of the investigation	Comments
Measles	Schatzmayr et al. (1982)	Seroconversion (SC) to measles vaccine by age in population of low social and economic status.	Supported the beginning of measles vaccination at nine months of age.
Measles	Oliva et al. (1986)	Immune response to measles CAM-70 vaccine in 341 children from six-12 months of age.	Measles CAM-70 vaccine seroconverted by neutralisation 100% of children = 9 months of age without maternal antibodies.
Measles	Camacho et al. (2000)	Dose-response to measles CAM-70 vaccine in children, three vaccine groups, with 5,000, 1,000 and 200 50% tissue culture infective dose (TCID_50_).	The group of higher dose, 5,000 TCID_50_ had better immunological response: 82% SC.
Measles	Lindgren-Alves et al. (2001)	Immune response to vaccine CAM-70 in 50 children, 21 perinatally infected with human immunodeficiency virus (HIV).	The immune response to measles vaccine is lower in HIV perinatally infected children, even after two doses.
Measles-mumps-rubella (MMR)	Boaventura et al. (2006)	To compare the immune response to three different MMR vaccines in schoolchildren in the state of Rio Grande do Sul, Brazil.	Satisfactory immune response to the three vaccines, but the Leningrad-Zagreb mumps component seems to be a stronger immunogen.
Oral poliomyelitis (OPV)	Schatzmayr and Homma (1969)	Immune response to three doses of OPV in 114 children from three months-three years of age in a semi-rural area of the state of Rio de Janeiro, Brazil.	SC to OPV types 1 and 3 was not satisfactory; type 1 dose should be increased. Vaccine in study had 500,000 TCID_50 _(type 1), 200,000 (type 2) and 300,000 (type 3) per dose.
OPV	Schatzmayr et al. (1986)	To compare the immune response between OPV and inactivated poliomyelitis vaccine (IPV) in 155 children at two-six months of age.	IPV induces more serum antibodies, especially to type 3.
OPV	Patriarca et al. (1988)	Immune response to trivalent OPV (TVOP) with increased type 3 dose compared with previous formulation.	TVOP induces higher SC to type 3 with formulation containing double the dose of type 3 (600,000 viral particles).
Hepatitis B (HB)	Motta et al. (2002)	To compare the immunogenicity of HB vaccine between preterm and term newborns.	Preterm newborns need an additional dose of HB vaccine.
HB	Martins et al. (2004)	Safety and immune response to recombinant HB vaccine from Butantan Institute, state of São Paulo, Brazil.	The HB vaccine from Butantan Institute is equivalent to Engerix B from GlaxoSmithKline (GSK) in children and inferior, but acceptable for use in newborns, adolescents and young adults.
HB	Luna et al. (2009)	Safety and immune response of newborns to the HB vaccine from Butantan Institute.	The HB vaccine from Butantan Institute is equivalent to Engerix B from GSK in newborns.
HB	Moraes et al. (2010)	Safety and immune response of adults to HB vaccine from Butantan Institute.	The HB vaccine from Butantan Institute is equivalent to Engerix B from GSK in adults from 31-40 years.
HB	Potsch et al. (2010)	To investigate the immune response to HB vaccine in HIV-infected adults, with double the routine dose and in four doses.	The immune response to HB vaccine is satisfactory with the new schedule.
HB	Motta-Castro et al. (2009)	Adherence to vaccination and immune response to HB vaccine in quilombo communities in central Brazil.	Low adherence to vaccination schedule and lower immune response in males and people = 40 years of age.
HB	Potsch et al. (2012)	Immune response to HB vaccine in HIV-infected adults with double the routine dose and in four doses.	The immune response to HB vaccine is satisfactory with the new schedule.
Rotavirus	Mascarenhas et al. (2002a)	Reinfections by rotavirus in children vaccinated with rhesus-human vaccine.	Reinfections occur and may be severe.
Rotavirus	Mascarenhas et al. (2002b)	Occurrence of rotavirus genotypes during clinical study with rhesus-human vaccine.	Many serotypes causing infections. P[8], G1, P[4] and G2 were found in 53% and 26.6% of infections.
Influenza	Santini-Oliveira et al. (2012)	Immune response of HIV-infected adults to H1N1 pandemic influenza vaccine with adjuvant in two single or two double doses.	HIV-infected persons have a better immune response to H1N1 influenza pandemic adjuvanted vaccine after two doses and double the routine dose.
Quadrivalent papillomavirus (qHPV)	Giuliano et al. (2011)	Prevention of infection and lesions by vaccine types in heterosexual men and in men who have sex with men.	HPV is effective for the prevention of infections and lesions by papillomavirus in heterosexual men and in men who have sex with men.
qHPV	Moreira Jr et al. (2011)	Safety and adverse events following qHPV vaccine.	qHPV vaccine has low reactogenicity and is safe.
qHPV	Palefsky et al. (2011)	Efficacy of qHPV vaccine against anal intraepithelial neoplasia in men who have sex with men.	qHPV vaccine reduces rates of anal intraepithelial neoplasia in men who have sex with men.
qHPV	Hillman et al. (2012)	Immune response to qHPV vaccine.	qHPV vaccine induces immune responses in 97.4% of men; immune responses in heterosexual men are higher than in men who have sex with men.

Source: Martins (2014).

**Table:** TABLE III Synopsis of studies with bacterial vaccines in chronological order of publication

Vaccine	Reference	Rationale of the investigation	Comments
Bacillus Calmette-Guérin (BCG)	Camacho and Klein (1990)	To use BCG and purified protein derivative as a tool to evaluate prevalence of tuberculosis (TB) infections.	The prevalence of TB infections in areas of the state of Rio de Janeiro, Brazil, was 4.13%.
BCG	Barbosa et al. (2003)	Cytokines immune response to BCG revaccination in schoolchildren.	Production of interferon (IFN)-γ increases after BCG revaccination and may be a marker of protection against TB.
BCG	Oliveira et al. (2013)	Production of IFN-γ after BCG revaccination.	In the group with IFN-γ production increases of 3.3 or more after BCG revaccination the immune responses to mycobacterial antigens remained higher one year after vaccination than on the group with lower IFN-γ increases.
Diphtheria, tetanus and pertussis/*Haemophilus influenzae* Type b (DTP/Hib)	Clemens et al. (2003)	To investigate the possibility of mixing the conjugate, lyophilised, Hib vaccine from GlaxoSmithKline (GSK) with the DTP/whole cell pertussis (DTPw) vaccine from Butantan Institute, state of São Paulo, Brazil.	The Hib conjugate, lyophilised, vaccine from GSK, may be mixed with the DTPw vaccine from Butantan Institute.
DTP/Hib	Martins et al. (2008b)	Consistency of production and non-inferiority of DTPw/Hib vaccine from Bio-Manguinhos/Oswaldo Cruz Foundation-Butantan Institute in comparison with DTPw/Hib vaccine with the DTP component from Butantan Institute and the Hib component from GSK.	Production of vaccine was consistent and the DTPw/Hib vaccine from Bio-Manguinhos/Butantan Institute is non-inferior to DTPw/Hib vaccine with the DTP component from Butantan Institute and the Hib component from GSK.
DTP/Hib	Matos et al. (2009)	Functional evaluation of immune response to poli-ribosil-ribitol-phosphate (PRP) from DTPw/Hib vaccine locally produced.	The immune response is mainly IgG1 and PRP avidity increases during vaccination, reaching maximum levels after the third dose.

Source: Martins (2014).

**Table:** TABLE IV Synopsis of studies with parasitic vaccines in chronological order of publication

Vaccine	Reference	Rationale of the investigation	Comments
Malaria	Urdaneta et al. (1996)	Adverse events after SPf66 malaria vaccine in Brazil.	The malaria SPf66 vaccine is safe.
Malaria	Urdaneta et al. (1998)	Efficacy of malaria SPf66 vaccine in endemic area.	There was no evidence of significant protection after SPf66 malaria vaccine against *Plasmodium falciparum* and *Plasmodium vivax*.
Leishmania	Mendonça et al. (1995)	Immunogenicity of *Leishmania* pentavalent vaccine in two doses of 360 µg with seven days interval.	Seventy-four percent of volunteers became positive on Montenegro test after vaccination; cellular immunity developed only for the tegumentary disease, not for the visceral disease.
Leishmania	Marzochi et al. (1998)	Immunogenicity and safety of *Leishmania* monovalent vaccine in one or two doses of 1.440 mg.	Induration area on Montenegro test was larger on the vaccinal groups than on placebo; vaccine is safe.
Leishmania	De Luca et al. (1999)	To compare immunogenicity of autoclaved and not autoclaved monovalent *Leishmania* vaccine.	Conversion, evaluated by the leishmanin skin-test (LST), was higher on the non autoclaved group than on the autoclaved group.
Leishmania	De Luca et al. (2001)	Immunogenicity of monovalent *Leishmania* vaccine in doses of 180, 360 and 540 µg, in two or three doses.	Conversion by LST test was 89.5% on the vaccine groups and 0% on the placebo group. Immunogenicity was higher on the group with 360 µg and two doses had immunogenicity similar to three doses.
*Necator americanus* (*Na-*ASP-2)	Diemert et al. (2012)	Safety and immune responses to *Na-*ASP-2 vaccine.	The *Na-*ASP-2 vaccine induced allergic reactions when given in an endemic area and study was interrupted.

Source: Martins (2014).

**Table:** TABLE V Number of clinical studies by type of vaccine

Viral vaccines	Studies(n)	Licensed for use
Yellow fever	26	All
Hepatitis B	7	
Quadrivalent papillomavirus	4	
Oral poliomyelitis	3	
Measles	4	
Measles/mumps/rubella	1	
Rotavirus	2	
Influenza	1	
Total	48	
Bacterial vaccines		
Bacillus Calmette-Guérin	3	All
Diphtheria, tetanus and pertussis/*Haemophilus influenzae* Type b (tetravalent)	3	
Total	6	
Parasitic vaccines		
Leishmania	4	None
Malaria	2	
Necator americanus	1	
Total	7	
All clinical studies	61	54/61 (88.5%)

Source: Martins (2014).

## DISCUSSION

The clinical studies with vaccines under the scope of IOC and Fiocruz, besides their relevance to the public health of Brazil and many other developing countries, have contributed to the institutional TD.

These technical advances built institutional knowledge and skills in vaccine thermostability, new freeze-drying formulation, use of certified inputs, improvement of quality control methodologies and the skills to incorporate new products through technology transfers, which resulted in scientific breakthroughs and have been landmarks of Fiocruz history.

Examples of tech transfer include the yellow fever (YF) vaccine (Rockefeller Institute), the polysaccharidic AC meningococcal vaccine (Institut Mérieux), the poliomyelitis and measles vaccines (BIKEN Institute), the *Haemophilus influenzae* *Type b* (*Hib*) vaccine, measles/mumps/rubella (MMR) and rotavirus vaccines [*GlaxoSmithKline (*GSK)] and shortly the measles/mumps/rubella/varicella (MMRV) vaccine (also with GSK). Besides leading to the introduction of new vaccines into the NIP, in a relatively short time, technology transfers of these vaccines have made possible the creation, expansion and improvement of new platforms, production and laboratories and the creation of a qualified workforce that is now a most valuable asset of Fiocruz. The positive consequences of these innovative processes have been outstanding and should not be minimised. Moreover, transfer of technologies has been feasible because of the intrinsic capacity of the institution for absorbing, in a relatively short span of time, the newly involved technologies.

The clinical studies of measles vaccines were conducted to evaluate the successful technology transfer and implement the regular use of a vaccine to counteract one of the main causes of child mortality in Brazil (Puffer & Serrano 1973). The technology of production of this vaccine was obtained thanks to the Brazil-Japan Cooperation Agreement, which involved the participation of BIKEN Laboratory from Osaka University, with Japan International Cooperation Agency and Funding Authority for Studies and Projects as intermediaries. This agreement made possible the development of new projects, which included: (i) an improvement and adaptation of laboratories for the production of viral antigens and formulation, filling and lyophilisation in industrial scale, (ii) the provision of industrial equipment, (iii) "on the bench" training in Japan and the beginning of production operations and (iv) the clinical studies in the states of Pernambuco and Pará. This project gave Bio-Manguinhos the opportunity to build the infrastructure for industrial production to meet today's good manufacturing practices requirements. This required new organisational structures including independent departments for production, quality control and management which, in a step by step process, resulted in the provision of good quality vaccines for the NIP. Because of this successful activity, Bio-Manguinhos is now prepared to provide the MMR and MMRV vaccines to the NIP.

The clinical studies with poliomyelitis vaccines and many additional seroepidemiological studies resulted in changes and improvements in vaccine composition and eventually resulted in the elimination of this disease in Brazil and many other Latin American countries.

As with the measles vaccine, the *oral polio vaccine* (OPV) technology was obtained under the umbrella of the Brazil-Japan Cooperation Agreement. The technology transfer for this vaccine came through the Japanese Poliomyelitis Research Institute. These actions included redesigns and upgrades of facilities, provision of equipment, "on the bench" training within production laboratories, quality control and neurovirulence testing in nonhuman primates. The creation of this highly qualified group in 1982 allowed Bio-Manguinhos to take the responsibility for the quality control testing of the OPV vaccine used in the national routine immunisation program or in mass campaigns. Later, this responsibility was transferred to the National Institute for Quality Control. Another great contribution from Bio-Manguinhos was the formulation improvements of OPV vaccine, including a doubling of the dose of type 3 OPV, required to control poliomyelitis outbreaks in Northeast Brazil. This formulation change was accomplished in just two weeks after the decision was made. The highly satisfactory results led the PAHO to extend this recommendation to all Latin American countries and, afterwards, WHO recommended the use of the same formulation for all countries.

The clinical studies with diphtheria, tetanus and pertussis/Hib vaccine drove the technology transfer for the Hib portion of the vaccine. This resulted in the introduction of this vaccine into the routine vaccination schedule, which, in a very short time, drastically reduced the incidence of Hib meningitis.

Table V shows that the clinical studies with viral and bacterial vaccines led to the licensing of these essential vaccines. Almost half these studies were conducted with YF vaccine - a demonstration of the importance of this vaccine for Brazil and the world and the need to improve it, in order to reduce its serious adverse events.

In contrast to the other clinical studies, the ones for parasitic diseases have not led to the licensure of any vaccine. Due to genetic variation of parasites, the epitope multiplicity and the complexity of anti-infectious mechanisms that are very different from virus and bacteria, it has been a big challenge to develop vaccines for parasitic diseases. Here, there is a need for new and innovative approaches. For malaria, there have been attempts to block transmission from the mosquito using vaccines targeting the sexual stages of *Plasmodium falciparum *(Biswas et al. 2013). In the case of leishmaniasis, there may be a combination of strategies, for example, antigens in nanoparticles (Santos et al. 2013) or a combination of treatment and immunotherapy (Machado-Pinto et al. 2002, Nascimento et al. 2010). Perhaps the traditional approach for highly effective preventive vaccines will have to be modified to include more modest but even so important objectives, such as disease attenuation or disease blocking transmission strategies.

The difficulties for the development of vaccines for some genetically unstable viruses, such as human immunodeficiency virus and hepatitis C, underscore the need to explore new technologies. These would include the presentation of antigens in virosomes and nanoparticles, chimeric vaccines, new adjuvants and new ways for delivering vaccines, such as in patches or microneedles. Other vaccines need improvement, such as for tuberculosis (TB), which needs better protection, and for pertussis and YF, which need improved safety.

Although some of the achievements have been remarkable, we should recognise that innovations at IOC and Fiocruz have been incremental and did not change paradigms (Kuhn 1970). The innovation which results in technological leaps is a process that begins many years before. It involves many groups working in cooperation on many different fields - microbiology, immunology, biochemistry, genetics. It requires the engagement of a critical mass of technological and human resources, a long term financial support and strong coordination and management.

The results presented here indicate that, over the last several years, there have been considerable quantitative and qualitative advances in the development of clinical studies under the scope of IOC and Fiocruz. These demonstrate that clinical studies no longer constitute a bottleneck for innovation, in terms of local capacity. However, regulatory and financial constraints still remain. There are also regulatory and operational issues that need to be addressed, in order to streamline processes without loss of safety and quality. Slow decision-making and excessive centralisation of regulatory and ethical processes may, in fact, decrease the quality of studies and may result in loss of opportunities in a competitive world.

It should be noted that, although not reviewed here, several clinical studies with meningococcal vaccines have been conducted at Bio-Manguinhos/Fiocruz, of which three have been Phase I studies and published in congress annals (Martins et al. 2009a, b, 2010).

Although several new innovative vaccines are in development, their complexity is increasing and will certainly require creativity and scientific and technological capacity to achieve a final product.

It should also be stressed that if we have been weak in technological innovation so far in Brazil, we have been innovators on the ethical concept that vaccines are a right of citizenship and that all people should have free access to them, as to basic sanitary services and education. The vaccine schedule of the Ministry of Health has significantly expanded and now includes all vaccines used by developed countries (Martins et al. 2013a). Moreover, there is a clear perception that vaccines have an excellent cost/benefit relationship and that they contribute to economic development (Bloom et al. 2005).

The contribution of vaccines for the improvement of health conditions of the Brazilian population is evident and outstanding. The infectious diseases targeted by vaccination (except TB) are under control. The Brazilian producers of vaccines made these conquests possible and the clinical studies with vaccines have been an essential part of this process.

The review of clinical studies under the scope of IOC and Fiocruz within the period of this study indicates the strength and potential of IOC and Fiocruz to conduct all phases of clinical studies with vaccines. However, the innovation component is still weak and should be strengthened. To achieve this objective as well as to accelerate the TD of new and innovative vaccines, we suggest: (i) the urgent development of a new legal and institutional framework for Bio-Manguinhos/Fiocruz, allowing flexibility and capacity to produce and to operate in the market. It is necessary to conciliate social commitment with speeding up of processes and entrepreneurial capacity, which are essential to industrial activity and technological competitiveness; (ii) to stimulate intra and inter institutional partnerships and the exchange of personnel, nationally and internationally; (iii) to stimulate group and personal cooperation through meetings and common projects; (iv) to promote public-private partnerships, leading to technology transfers, to search for common development of new products and processes; (v) to stimulate innovation and creativity; (vi) to attract new talent, creating an inspiring and receptive atmosphere and environment to innovation and creativity; (vii) to search for expertise, wherever it may reside, to solve the technological and organisational problems of Bio-Manguinhos/Fiocruz; (viii) to increase the governmental financing for clinical studies and to stimulate the non-governmental financing; (ix) to stimulate excellence, at all levels.

## References

[B1] Artenstein AW (2010). Vaccines, a biography, Springer.

[B2] Barbosa T, Arruda S, Fernandes BD, Carvalho LP, Cardoso S, Cunha S, Barreto ML, Pereira SM, Rodrigues LC, Barral-Netto M (2003). BCG (Bacille of Calmette-Guérin) revaccination leads to improved in
vitro IFN-γ response to mycobacterial antigen independent of tuberculin
sensitization in Brazilian school-age children. Vaccine 21.

[B3] Benchimol JL (2001). Febre amarela - a doença e a vacina, uma história inacabada.

[B4] Biswas S, Li Y, Miura K, Zakutansky SE, Long CA, Sinden RE, Draper SJ, Hill F (2013). Enhancing antibody immunogenicity of transmission-blocking malaria
vaccines. Am J Trop Med Hyg.

[B5] Bloom DE, Canning D, Weston M (2005). The value of vaccination. World Economics.

[B6] Boaventura AS, Stralioto SM, Siqueira MM, Ranieri TS, Bercini M, Schermann MT, Wagner MB, Silveira TR (2006). Prevalence of antibodies against measles, mumps and rubella before and
after vaccination of school-age children with three different triple combined
viral vaccines, Rio Grande do Sul, Brazil. Rev Panam Salud Publica.

[B7] Camacho LAB, Aguiar SG, Nascimento JP, Freire MS, Leal MLF, Iguchi T, Lozana JA, Farias RHG, Grupo Colaborativo para o Estudo das Vacinas de Febre Amarela
2005 (2005). Reactogenicity of yellow fever vaccines in a randomized,
placebo-controlled trial. Rev Saude Publica.

[B8] Camacho LAB, Freire MS, Leal MLF, Aguiar SG, Nascimento JP, Iguchi T, Lozana JA, Farias RHG, Grupo Colaborativo para o Estudo das Vacinas de Febre Amarela
2004 (2004). Immunogenicity of WHO-17D and Brazilian 17DD yellow fever vaccines: a
randomized trial. Rev Saude Publica.

[B9] Camacho LAB, Freire MS, Yamamura AMY, Leal ML, Mann G (2000). Estudo de soroconversão com formulações da vacina BIKEN CAM-70 contra
sarampo. Rev Saude Publica.

[B10] Camacho LAB, Klein CH (1990). Risco de infecção tuberculosa entre escolares com alta cobertura pelo
BCG. Bol Oficina Sanit Panam.

[B11] Campi-Azevedo AC, Araujo-Porto LP, Luiza-Silva M, Batista MA, Martins MA, Sathler AR, Silveira-Lemos D, Camacho LAB, Martins RM, Maia MLS, Farias RHG, Freire MS, Galler R, Homma A, Ribeiro JGL, Lemos JAC, Auxiliadora-Martins M, Caldas IR, Elói-Santos SM, Teixeira-Carvalho A, Martins-Filho O (2012). 17DD and 17D-213/77 yellow fever substrains trigger a balanced
cytokine profile in primary vaccinated children. PLoS ONE.

[B12] Clemens SA, Azevedo T, Homma A (2003). Feasibility study of the immunogenicity and safety of a novel DTPw/Hib
(PRP-T) Brazilian combination compared to a licensed vaccine in healthy children
at 2, 4 and 6 months of age. Rev Soc Bras Med Trop.

[B13] De Luca PM, Mayrink W, Alves CR, Coutinho SG, Oliveira MP, Bertho AL, Toledo VP, Costa CA, Genaro O, Mendonça CF (1999). Evaluation of the stability and immunogenicity of autoclaved and
nonautoclaved preparations of a vaccine against American tegumentary
leishmaniasis. Vaccine.

[B14] De Luca PM, Mayrink W, Pinto JA, Coutinho SG, Santiago MA, Toledo VP, Costa CA, Genaro O, Reis AB, Mendonça SCF (2001). A randomized double-blind placebo-controlled trial to evaluate the
immunogenicity of a candidate vaccine against American tegumentary
leishmaniasis. Acta Trop.

[B15] Diemert DJ, Pinto AG, Freire J, Jariwala A, Santiago H, Hamilton RG, Periago MV, Loukas A, Tribolet L, Mulvenna J, Correa-Oliveira R, Hotez P, Bethony JM (2012). Generalized urticaria induced by theNa-ASP-2 hookworm vaccine:
implications for the development of vaccines against helminths. J Allergy Clin Immunol.

[B16] dos Santos AP, Bertho AL, Dias DC, Santos JR, Marcovistz R (2005). Lymphocyte subset analyses in healthy adults vaccinated with yellow
fever 17DD virus. Mem Inst Oswaldo Cruz.

[B17] dos Santos AP, Bertho AL, Martins RM, Marcovistz R (2007). The sample processing time interval as an influential fator in flow
cytometry analysis of lymphocyte subsets. Mem Inst Oswaldo Cruz.

[B18] Fox JP, Cabral AS (1943). The duration of immunity following vaccination with the 17D strain of
yellow fever virus. Am J Hyg.

[B19] Fox JP, Cunha JF, Kossobudzki SL (1948). Additional observations on the duration of immunity following
vaccination with the 17D strain of yellow fever virus. Am J Hyg.

[B20] Fox JP, Kossobudzki SL, Cunha JF (1943). Field studies on the immune response to 17D yellow fever
virus. Am J Hyg.

[B21] Fox JP, Lennette EH, Manso C, Aguiar JRS (1942). Encephalitis in man following vaccination with 17D yellow fever
virus. Am J Hyg.

[B22] Fox JP, Manso C, Penna HA, Pará M (1942). Observations on the occurrence of icterus in Brazil following
vaccination against yellow fever. Am J Hyg.

[B23] Freire MS, Carvalho R, Yamamura AMY, Almeida LFC, Santos HHL, Borges MBJ, Mann GF (2002). Seroconversion following vaccination with the 17DD substrain of yellow
fever virus. Virus Rev Res.

[B24] Giuliano AR, Palefsky JM, Goldstone S, Moreira ED, Penny ME, Aranda C, Vardas E, Moi H, Jessen H, Hillman R, Chang Y, Ferris D, Rouleau D, Bryan J, Marshall JB, Vuocolo S, Barra E, Radley D, Haupt RM, Guris D (2011). Efficacy of quadrivalent HPV vaccine against HPV infection and disease
in males. N Engl J Med.

[B25] Groot H, Ribeiro RB (1962). Neutralizing and haemagglutination-inhibiting antibodies to yellow
fever 17 years after vaccination with 17D vaccine. Bull World Health Organ.

[B26] Hillman RJ, Giuliano AR, Palefsky JM, Goldstone S, Moreira ED, Vardas E, Aranda C, Jessen H, Ferris DG, Coutlee F, Marshall JB, Vuocolo S, Haupt RM, Guris D, Garner EIO (2012). Immunogenicity of the quadrivalent human papillomavirus (type
6/11/16/18) vaccine in males 16 to 26 years old. Clin Vaccine Immunol.

[B27] Kuhn TS (1970). The structure of scientific revolutions.

[B28] Lindgren-Alves CR, Freire LMS, Oliveira RC, Guerra HL, Da-Silva EE, Siqueira MM, Horta IM, Queiroz CC (2001). Pesquisa de anticorpos contra o sarampo em crianças infectadas pelo
HIV após imunização básica. J Pediatr (Rio J).

[B29] Lopes OS, Guimarães SSDA, Carvalho R (1988). Studies on yellow fever vaccine. III - Dose response in
volunteers. J Biol Stand.

[B30] Luiza-Silva M, Campi-Azevedo AC, Batista MA, Martins MA, Avelar RS, Lemos DS, Camacho LAB, Martins RM, Maia MLS, Farias RHG, Freire MS, Galler R, Homma A, Ribeiro JGL, Lemos JAC, Martins MA, Eloi-Santos SM, Teixeira-Carvalho A, Martins-Filho OA (2011). Cytokine signatures of innate and adaptive immunity in 17DD yellow
fever vaccinated children and its association with the level of neutralizing
antibody. J Infect Dis.

[B31] Luiza-Silva M, Martins MA, Espírito-Santo LR, Campi-Azevedo AC, Silveira-Lemos D, Ribeiro JGL, Homma A, Kroon EG, Teixeira-Carvalho A, Eloi-Santos SM, Martins-Filho OA (2011). Characterization of main cytokine sources from the innate and adaptive
imune responses following primary 17DD yellow fever vaccination in
adults. Vaccine.

[B32] Luna EJ, Moraes JC, Silveira L, Salina HSN (2009). Efficacy and safety of the Brazilian vaccine against hepatitis B in
newborns. Rev Saude Publica.

[B33] Machado-Pinto J, Pinto J, da Costa CA, Genaro O, Marques MJ, Modabber F, Mayrink W (2002). Immunochemotherapy for cutaneous leishmaniasis: a controlled trial
using killedLeishmania amazo- nensisvaccine plus antimonial. Int J Dermatol.

[B34] Martins MA, Silva ML, Elói-Santos SM, Ribeiro JGL, Peruhype-Magalhães V, Marciano APV, Homma A, Kroon EG, Teixeira-Carvalho A, Martins-Filho OA (2008). Innate immunity phenotypic features point toward simultaneous raise of
activation and modulation events following 17DD live attenuated yellow fever
first-time vaccination. Vaccine.

[B35] Martins MA, Silva ML, Marciano APV, Peruhype-Magalhães V, Eloi-Santos SM, Ribeiro JGL, Correa-Oliveira R, Homma A, Kroon EG, Teixeira-Carvalho A, Martins-Filho OA (2007). Activation/modulation of adaptive immunity emerges simultaneously
after 17DD yellow fever first-time vaccination: is this the key to prevent severe
adverse reactions following immunization?. Clin Exp Immunol.

[B36] Martins RM (2014). Análises de estudos clínicos com vacinas realizados no âmbito do Instituto
Oswaldo Cruz e Fiocruz - memória, avaliação e lições.

[B37] Martins RM, Barbosa GG, Maia MLS, Engstrom E, Camacho LAB, Périssé AR, Silveira IAFB, Leal ML, Marcovistz R, Homma A, Jessouroun E (2010). Phase I study of a meningococcal C strain 2135 conjugate vaccine.

[B38] Martins RM, Bensabath G, Arraes LC, Oliveira MLA, Miguel JC, Barbosa GG, Camacho LAB (2004). Multicenter study on the immunogenicity and safety of two recombinant
vaccines against hepatitis B. Mem Inst Oswaldo Cruz.

[B39] Martins RM, Camacho LAB, Marcovistz R, de Noronha TG, Maia MLS, dos Santos EM, Barbosa GG, da Silva AMV, de Souza PCNF, Lemos MCF, Homma A (2008). Immunogenicity, reactogenicity and consistency of production of a
Brazilian combined vaccine against diphtheria, tetanus, pertussis andHaemophilus
influenzaeType b. Mem Inst Oswaldo Cruz.

[B40] Martins RM, Camacho LAB, Périssé AR, Santos TM, Silveira IAFB, Maia MLS, Marcovistz R, Maia MLS, Homma A, Jessouroun E (2009). Phase I safety and immunogenicity study of a serogroup C conjugate vaccine -
Lessons learned.

[B41] Martins RM, Homma A, Migowski E, Coura JR (2013). Imunizações. Dinâmica das doenças infecciosas e parasitárias.

[B42] Martins RM, Maia MLS, Farias RHG, Camacho LAB, Freire MS, Galler R, Yamamura AMY, Almeida LFC, Lima SMB, Nogueira RMR, Sá GRS, Hokama DA, Carvalho R, Freire RAV, Pereira E, Leal MLF, Homma A (2013). 17DD yellow fever vaccine. A Double blind, randomized clinical trial
of immunogenicity and safety on a dose-response study. Hum Vaccin Immunother.

[B43] Martins RM, Périssé AR, Camacho LAB, Santos TM, Silveira IAFB, Leal ML, Maia MLS, Homma A, Jessouroun E (2009). Phase I safety and immunogenicity study of a Brazilian bivalent
serogroup B vaccine. Neisseria vaccines 2009.

[B44] Marzochi KBF, Marzochi MCA, Silva AF, Grativol N, Duarte R, Confort EM, Modabber F (1998). Phase 1 study of an inactivated vaccine against American tegumentary
leishmaniasis in normal volunteers in Brazil. Mem Inst Oswaldo Cruz.

[B45] Mascarenhas JDP, Leite JPG, Gabbay YB, Freitas RB, Oliveira CS, Monteiro TAF, Linhares AC (2002). Rotavirus G serotypes and P[], G genotypes identified in cases of
reinfection among children participating in a trial with rhesus-human reassortant
tetravalent vaccine (RRV-TV) in Belém, Brazil. J Trop Pediatr.

[B46] Mascarenhas JDP, Linhares AC, Gabbay YB, Leite JPG (2002). Detection and characterization of rotavirus G and P types from
children participating in a rotavirus vaccine trial in Belém,
Brazil. Mem Inst Oswaldo Cruz.

[B47] Matos DCS, Silva AMV, Neves PCC, Martins RM, Homma A, Marcovistz R (2009). Pattern of functional antibody activity againstHaemophilus
influenzaeType b (Hib) in infants immunized with diphtheria-tetanus-pertussis/Hib
Brazilian combination vaccine. Braz J Med Biol Res.

[B48] Melo AB, Nascimento EJM, Braga-Neto U, Dhalia R, Silva AM, Oelke M, Schneck JP, Sidney J, Sette A, Montenegro SML, Marques ET (2013). T-cell memory responses elicited by yellow fever vaccine are targeted
to overlapping epitopes containing multiple HLA-I and II binding
motifs. PLoS Negl Trop Dis.

[B49] Melo AB, Silva MPC, Magalhães MCF, Gil LHV, Carvalho EMF, Braga-Neto UM, Bertani GR, Marques ETA, Cordeiro MT (2011). Description of a prospective 17DD yellow fever vaccine cohort in
Recife, Brazil. Am J Trop Med Hyg.

[B50] Mendonça SCF, De Luca PM, Mayrink W, Restom TG, Conceiçao-Silva F, Da-Cruz AM, Bertho AL, Costa CA, Genaro O, Toledo VPCP, Coutinho SG (1995). Characterization of human T lymphocyte-mediated immune responses
induced by a vaccine against American tegumentary leishmaniasis. Am J Trop Med Hyg.

[B51] Moraes JC, Luna EJA, Grimaldi RA (2010). Immunogenicity of the Brazilian hepatitis B vaccine in
adults. Rev Saude Publica.

[B52] Moreira ED, Palefsky JM, Giuliano AR, Goldstone S, Aranda C, Jessen H, Hillman RJ, Ferris D, Coutlee F, Vardas E, Marshall JB, Vuocolo S, Haupt RM, Guris D, Garner EIO (2011). Safety and reactogenicity of a quadrivalent human papillomavirus
(types 6, 11, 16, 18) L1 viral-like-particle vaccine in older adolescentes and
young adults. Hum Vaccin.

[B53] Motta MSF, Mussi-Pinhata MM, Jorge SM, Yoshida CFT, Souza CBS (2002). Immunogenicity of hepatitis B vaccine in preterm and full term infants
vaccinated within the first week of life. Vaccine.

[B54] Motta-Castro ARC, Gomes SA, Yoshida CFT, Miguel JC, Teles SA, Martins RMB (2009). Compliance with and response to hepatitis B vaccination in remaining
quilombo communities in central Brazil. Cad Saude Publica.

[B55] Nascimento E, Fernandes DF, Vieira EP, Campos-Neto A, Ashman JA, Alves FP, Coler RN, Bogatzki LY, Kahn SJ, Beckmann AM, Pine SO, Cowgill KD, Reed SG, Piazza FM (2010). A clinical trial to evaluate the safety and immunogenicity of the
LEISH-F1+MPL-SE vaccine when used in combination with meglumine antimoniate for
the treatment of cutaneous leishmaniasis. Vaccine.

[B56] Neves PCC, Matos DCS, Marcovistz R, Galler R (2009). TLR expression and NK activation after human yellow fever
vaccination. Vaccine.

[B57] Oliva OP, Chaves JRS, Loureiro MLP, Pereira LA, Homma A (1986). Vacina CAM-70 contra o sarampo produzida no Brasil. Avaliações de
campo em crianças com 6-12 meses de idade. Boletim Epidemiológico.

[B58] Oliveira ES, Marinho JM, Barbosa T (2013). Interferon-gamma production by mononuclear cells in Bacille
Calmette-Guérin-revaccinated healthy volunteers predicted long-term
antimycobacterial responses in a randomized controlled trial. Vaccine.

[B59] Palefsky JM, Giuliano AR, Goldstone S, Moreira ED, Aranda C, Jessen H, Hillman R, Ferris D, Coutlee F, Stoler MH, Marshall JB, Radley D, Vuocolo S, Haupt RM, Guris D, Garner EIO (2011). HPV vaccine against anal HPV infection and anal intraepithelial
neoplasia. N Engl J Med.

[B60] Patriarca PA, Palmeira G, Lima J, Cordeiro MT, Laender F, Oliveira MJC, Dantes MCS, Risi JB (1988). Randomised trial of alternative formulations of oral poliovaccine in
Brazil. Lancet.

[B61] Plotkin AS (2011). History of vaccine development.

[B62] Potsch DV, Camacho LAB, Tuboi S, Villar LM, Miguel JC, Ginuino C, Silva EF, Mendonça RMM, Moreira RB, Barroso PF (2012). Vaccination against hepatitis B with 4-double doses increases response
rates and antibodies titers in HIV-infected adults. Vaccine.

[B63] Potsch DV, Oliveira MLA, Ginuino C, Miguel JC, Oliveira SAN, Silva EF, Moreira RB, Cruz GVM, Oliveira ALVSM, Camacho LAB, Barroso PF (2010). High rates of serological response to a modified hepatitis B
vaccination schedule in HIV-infected subjects. Vaccine.

[B64] Puffer RR, Serrano CV (1973). Características de la mortalidad en la niñez: informe de la Investigación
Interamericana de la Niñez.

[B65] Santini-Oliveira M, Camacho LAB, Souza TML, Luz PM, Vasconcellos MTL, Giacoia-Gripp CBW, Morgado MG, Nunes EP, Lemos AS, Ferreira ACG, Moreira RI, Veloso VG, Siqueira MM, Grinsztejn B (2012). H1N1 pdm09 adjuvanted vaccination in HIV-infected adults: a randomized
trial of two singleversustwo double doses. PLoS ONE.

[B66] Santos AP, Matos DCS, Bertho AL, Mendonça SCF, Marcovistz R (2008). Detection of TH1/ TH2 cytokine signatures in yellow fever 17DD
first-time vaccinees through ELISpot assay. Cytokine.

[B67] Santos DM, Carneiro MW, de Moura TR, Soto M, Luz NF, Prates DB, Irache JM, Brodskyn C, Barral A, Barral-Netto M, Espuelas S, Borges VM, de Oliveira CI (2013). PLGA nanoparticles loaded with KMP-11 stimulate innate immunity and
induce the killing of Leishmania. Nanomedicine.

[B68] Schatzmayr HG, Homma A (1969). Avaliação sorológica da vacina oral, tipo Sabin, contra a
poliomielite, em região semi-rural. I. Formação de anticorpos em
vacinados. Rev Soc Bras Med Trop.

[B69] Schatzmayr HG, Maurice Y, Fujita M, Fillipis AMB (1986). Serological evaluation of poliomyelitis oral and inactivated vaccines
in an urban low-income population at Rio de Janeiro, Brazil. Vaccine.

[B70] Schatzmayr HG, Nogueira RMR, Bermudez JAZ, Pinhão AT, Queiroz B, Venâncio LR, Assis CER, Shiraiwa T (1982). Serological response to measles vaccine (Schwarz strain) in a
low-income population at Rio de Janeiro. Rev Microbiol.

[B71] Silva JRN, Camacho LAB, Siqueira MM, Freire M, Castro YP, Maia MLS, Yamamura AMY, Martins RM, Leal MLF, Grupo Colaborativo para o Estudo das Vacinas de Febre Amarela
2011 (2011). Mutual interference on the immune response to yellow fever vaccine and
a combined vaccine against measles, mumps and rubella. Vaccine.

[B72] Smith HH, Penna HA, Paoliello A (1938). Yellow fever vaccination with cultured virus (17D) without immune
serum. Am J Trop Med Hyg.

[B73] Soper FL, Smith HH (1938). Yellow fever vaccination with cultivated virus and immune and
hyperimmune serum. Am J Trop Med Hyg.

[B74] Stefano I, Sato HK, Pannuti CS, Omoto TM, Mann G, Freire MS, Yamamura AMY, Vasconcelos PFC, Oselka GW, Weckx LW, Salgado MF, Noale LFO, Souza VAUF (1999). Recent immunization against measles does not interfere with the
sero-response to yellow fever vaccine. Vaccine.

[B75] Urdaneta M, Prata A, Struchiner CJ, Tosta CE, Tauil P, Boulos M (1996). Safety evaluation of SPf66 malaria vaccine in Brazil. Rev Soc Bras Med Trop.

[B76] Urdaneta M, Prata A, Struchiner CJ, Tosta CE, Tauil P, Boulos M (1998). Evaluation of SPf66 malaria vaccine efficacy in Brazil. Am J Trop Med Hyg.

